# Assessment of the Phytotoxicity of Metal Oxide Nanoparticles on Two Crop Plants, Maize (*Zea mays* L.) and Rice (*Oryza sativa* L.)

**DOI:** 10.3390/ijerph121214963

**Published:** 2015-11-30

**Authors:** Zhongzhou Yang, Jing Chen, Runzhi Dou, Xiang Gao, Chuanbin Mao, Li Wang

**Affiliations:** 1 Institute of Genetics and Cytology, Northeast Normal University, Changchun 130024, China; yangzz121@nenu.edu.cn (Z.Y.); chenj316@nenu.edu.cn (J.C.); dourz288@nenu.edu.cn (R.D.); gaoxiang424@163.com (X.G.); 2 Key Laboratory of Molecular Epigenetics of MOE, Changchun 130024, China; 3 Department of Chemistry and Biochemistry, Stephenson Life Sciences Research Center, University of Oklahoma, Norman, OK 73019, USA

**Keywords:** phytotoxicity, metal oxide nanoparticles, maize, rice, germination

## Abstract

In this study, the phytotoxicity of seven metal oxide nanoparticles(NPs)—titanium dioxide (nTiO_2_), silicon dioxide (nSiO_2_), cerium dioxide (nCeO_2_), magnetite (nFe_3_O_4_), aluminum oxide (nAl_2_O_3_), zinc oxide (nZnO) and copper oxide (nCuO)—was assessed on two agriculturally significant crop plants (maize and rice). The results showed that seed germination was not affected by any of the seven metal oxide NPs. However, at the concentration of 2000 mg·L^−1^, the root elongation was significantly inhibited by nCuO (95.73% for maize and 97.28% for rice), nZnO (50.45% for maize and 66.75% for rice). On the contrary, minor phytotoxicity of nAl_2_O_3_ was only observed in maize, and no obvious toxic effects were found in the other four metal oxide NPs. By further study we found that the phytotoxic effects of nZnO, nAl_2_O_3_ and nCuO (25 to 2000 mg·L^−^^1^) were concentration dependent, and were not caused by the corresponding Cu^2+^, Zn^2+^ and Al^3+^ ions (0.11 mg·L^−^^1^, 1.27 mg·L^−^^1^ and 0.74 mg·L^−^^1^, respectively). Furthermore, ZnO NPs (<50 nm) showed greater toxicity than ZnO microparticles(MPs)(<5 μm) to root elongation of both maize and rice. Overall, this study provided valuable information for the application of engineered NPs in agriculture and the assessment of the potential environmental risks.

## 1. Introduction

The use of engineered nanoparticles (NPs) in medicine, cosmetics, energy, agriculture and machinery, *etc.* has increased rapidly [1,2]. Considering that engineered NPs can be released to the environment, accidentally or incidentally [3,4], nanotoxicity is receiving increasing attentions currently [5,6,7,8,9]. Despite the fact that more and more researchers have reported nanotoxicity in plants [10,11,12], the studies are still at the emerging stage and knowledge on the effects of NPs in plant systems needs further investigation [13,14], especially in crop plants, since the engineered NPs may be a threat to human health through the food chain [15].

Engineered NPs are divided into four categories according to USEPA: carbon-based materials, metal-based materials, dendrimers and composites. As an important part of metal-based materials, metal oxide NPs are widely used in industry, cosmetics, environmental pollution control, *etc.* [16,17]. At present, many kinds of metal oxide NPs have been applied in agriculture, specifically in plant protection and fertilization [18]. For instance, silicon dioxide NPs can be used as controlled release carriers in drug delivery and as an active ingredient against insect pests [19]; zinc oxide NPs can be used as insecticides [20]. 

Indeed, some metal oxide NPs were reported to have positive effects on crop plants. For example, the spraying of nTiO_2_ at the dose of 0.25‰~4‰ could significantly promote the growth of spinach [21]; root length of green peas treated with nZnO at 125, 250 and 500 mg·kg^−1^ soil was approximately two times longer than the control [11]. Nevertheless, more and more researchers have reported the phytotoxicity of metal oxide NPs in crop plants. For instance, Asli *et al.* reported that nTiO_2_ with mean diameters of 30 nm inhibited leaf growth and transpiration of maize seedlings [22]. Kim *et al.* proved that both 1000 mg·kg^−1^ nCuO and nZnO could significantly inhibit the growth of cucumber by 44%, respectively [23]. In this case, it can be seen that opposite conclusions (positive effects or negative effects) can be drawn about the same metal oxide NPs in different plants. More interestingly, the effects on the same plant caused by the same sort of metal oxide NPs also show totally opposite results. For instance, Castiglione *et al.* reported that nTiO_2_ at 4‰ could significantly inhibit the seed germination and root elongation of maize [24], while Burke *et al.* showed that nTiO_2_ at 200 mg·kg^−1^ had no significant effects on maize [12]. These opposite conclusions were probably caused by the different synthetic methods of the metal oxide NPs, different concentration and different media (hydroponics or soil culture), *etc.* Therefore, previous studies of the phytotoxicity of metal oxide NPs are confusing and somewhat controversial, due to the absence of integrated evaluation systems. In addition, the toxicity in crop plants was usually assessed using one or two kinds of metal oxide NPs. Therefore, it is urgent to systematically investigate the phytotoxicity of a wide variety of metal oxide NPs in crop plants.

Maize (*Zea mays* L.) is one of the three most important food crops worldwide. Rice (*Oryza sativa* L.), as another important staple food, feeding more than half of the World’s population [25]. Due to their important roles in the food security of humankind, the assessment of the phytotoxicity of metal oxide NPs on these two agriculturally important crop plants is crucial to human health. 

In the present study, germination experiments were carried out on maize and rice to evaluate the phytotoxicity of seven metal oxide NPs—titanium dioxide (nTiO_2_), silicon dioxide (nSiO_2_), cerium dioxide (nCeO_2_), magnetite (nFe_3_O_4_), aluminum oxide (nAl_2_O_3_), zinc oxide (nZnO) and copper oxide (nCuO). Root length and shoot length, which are sensitive to an adverse environment, were chosen as toxicity indicators. Based on the USEPA guidelines [26], a concentration of 2000 mg·L^−1^ was chosen as the preliminary concentration to assess the phytotoxicity of the seven metal oxide NPs. In addition, we also studied the effects of the exposure concentration and ion release on the phytotoxicity of nCuO, nZnO and nSiO_2_. The role of particle size in phytotoxicity was also determined for two different sizes of ZnO (50 nm, 5 μm). This study provided valuable information for the application of metal oxide NPs in agriculture and environmental safety assessment.

## 2. Experimental Section 

### 2.1. Nanomaterials and Seeds

All metal oxide NPs—nTiO_2_, nSiO_2_, nCeO_2_, nFe_3_O_4_, nAl_2_O_3_, nZnO, nCuO and ZnO microparticles (ZnO MPs, <5 μm)—were purchased from Sigma-Aldrich (St. Louis, MO, USA). The seeds of maize (Zhengdan No. 958) and rice (Jijing No. 6) were purchased from Seed Building of Changchun, China, and stored in a dry place at room temperature. Preliminary studies showed that the average germination rates of these seeds were higher than 95% to ensure the further treatment. 

### 2.2. Preparation and Characterization of Nanoparticle Suspensions 

The size and morphology were determined by transmission electron microscope (TEM) at 80 kV (H-7500, Hitachi, Ltd., Tokyo, Japan). For TEM observation, NP suspensions were prepared according to the following method: a certain quantity of metal oxide NPs were suspended in deionized water directly to make a concentration of 100 mg·L^−1^ and dispersed by ultrasonic vibration (100 W, 40 KHz) for 45 min. Then a drop of metal oxide NPs suspensions was dropped on the copper grid and dried at room temperature overnight. The zeta potential of the particles in the suspensions was estimated by a Zetasizer Nano ZS (Malvern Instruments, Malvern, UK). 

### 2.3. Measurement of the Content of the Dissolved Metal Ions 

To investigate the roles of the dissolved metal ions in causing phytotoxicity, total dissolved Cu^2+^, Zn^2+^ and Al^3+^ content of metal oxide NPs suspensions were determined. Firstly, nCuO, nZnO and nAl_2_O_3_ suspensions at 2000 mg·L^−1^ were centrifuged at 15,000 rpm for 30 min after dispersal by ultrasonic vibration (100 W, 40 KHz) for 45 min. Then, the supernatants were filtered through 0.2 μm glass filters, and the content of Cu, Zn and Al elements was analysed by an inductively coupled plasma optical emission spectrometer (ICP-OES, Prodigy, Leeman, Hudson, NH, USA). The wavelengths used were 327.396 nm, 213.856 nm and 309.271 nm for the elements Cu, Zn and Al, respectively.

### 2.4. Seed Germination

For germination, the seeds were surface sterilized by soaking in 70% ethanol for 2 min. Then the seeds were rinsed with sterile water several times to remove any remaining ethanol [27]. Subsequently, the seeds were immersed in sterile water or metal oxide NPs suspensions for 2 h. After that, every ten seeds were transferred into a Petri dish (100 mm × 15 mm) with a filter paper on the bottom, with the distance among each seeds ≥1 cm. Then 5 mL of test solution was added to each Petri dish. Finally, the Petri dishes were covered and sealed with tape. The germination was conducted in dark at 25 °C. After 5 days for maize and 7 days for rice, respectively, the germination rate, the root length and shoot length were measured [14,28]. 

### 2.5. Data Analysis 

Each treatment was conducted with three replicates and all of the experiment groups were conducted in triplicates, the dates were showed as mean ± SD (standard deviation). Difference analysis was conducted with ANOVA (analysis of variance, LSD). Statistical significance was based on probabilities of *p* ≤ 0.05.

## 3. Results and Discussion

### 3.1. Characterization of Metal Oxide NPs

**Figure 1:**
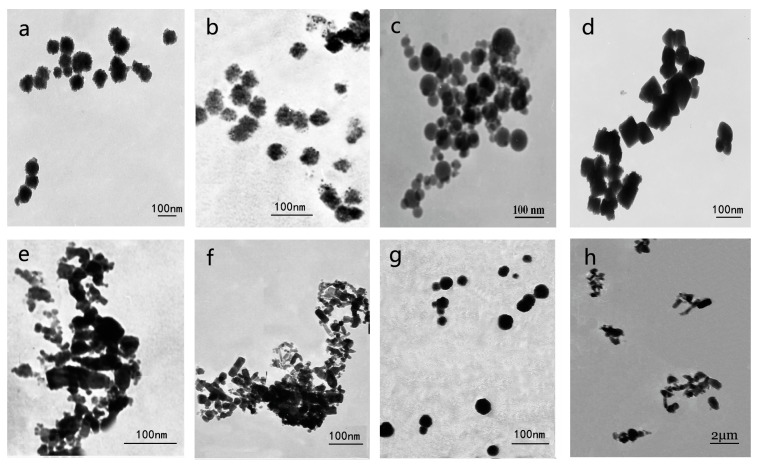
Transmission electron microscopy images of test materials. (**a**) nTiO_2_; (**b**) nSiO_2_; (**c**) nCeO_2_; (**d**) nFe_3_O_4_; (**e**) nAl_2_O_3_; (**f**) nZnO; (**g**) nCuO; (**h**) ZnO MPs.

Figure 1 shows TEM images of the seven metal oxide NPs and ZnO MPs, the size and morphology of which was almost the same as that given by the supplier. 

The data from both the producer and TEM are listed in Table 1. In addition, the purity of all the test particles provided by the supplier was included, except for nAl_2_O_3_. The zeta potential and pH values of seven metal oxide NPs and ZnO MPs suspensions (100 mg·L^−1^ for measuring zeta potential and 2000 mg·L^−1^ for measuring pH values) were also listed in Table 1.

**Table 1:** Test Material Information.

Particles	Size Sigma-Aldrich	Purity Sigma-Aldrich	Zeta Potential (mV)	pH	Description (TEM)
nTiO_2_	21 nm	99.5%	11.6 ± 1.1	7.13 ± 0.03	Spherical, present in the form of 60–120 nm irregular aggregate in water solution
nSiO_2_	5–15 nm (TEM)	99.5%	−17.6 ± 1.0	6.67 ± 0.03	Spherical, about 5–15 nm, usually gathered into 90 ± 30 nm irregular aggregate
nCeO_2_	<25 nm (BET)	99%	35.1 ± 0.7	6.71 ± 0.04	Spherical, with smooth edge and inhomogenous size, less than 50 nm
nFe_3_O_4_	50–100 nm (TEM)	97%	9.12 ± 0.47	7.08 ± 0.03	Keen-edged diamond or square, with the inhomogenous size of 50–100 nm
nAl_2_O_3_	13 nm (TEM)	-	29.5 ± 0.6	7.17 ± 0.02	Spherical, with the average size of 15 nm, easy to aggregate
nZnO	<50 nm (BET)	97%	−7.63 ± 0.37	7.14 ± 0.03	Clavate or irregular spherical, with inhomogenous size distribution, less than 50 nm
nCuO	<50 nm (TEM)	97%	20.6 ± 0.6	6.36 ± 0.02	Elliptic or spherical, with the size range of 40–80 nm
ZnO MPs	<5 µm	99.9%	10.4 ± 1.3	6.95 ± 0.03	Irregular shapes

### 3.2. Preliminary Assessment of the Phytotoxicity of Metal Oxide NPs

Concerns about the potential impacts of metal oxide NPs on plants have attracted increasing attention. However, the absence of systematic research methods and evaluation systems has lead to contradictory conclusions. In this study, a simple, rapid and sensitive germination experiment in Petri dishes was conducted to evaluate the phytotoxicity of metal oxide NPs. Germination is the beginning of the physiological process in the life of plants, and it is usually affected by various factors, like temperature, humidity, gas transfer, soil compaction, *etc.* [29]. In this experiment, the interferences were excluded as the seeds were directly contacted with metal oxide NPs. Both for maize and rice, the germination rates were not affected by the seven metal oxide NPs at 2000 mg·L^−1^. This result was consistent with previous studies, where seed germination rates were generally insensitive to NP exposure compared with the root elongation. For instance, nCuO, nCeO_2_ and nSiO_2_ have been reported to have no effect on germination rates, but significantly inhibit root elongation [9,30,31]. This result was probably due to the protection of the seed coat, which protects the seeds from the NPs [32].

**Figure 2:**
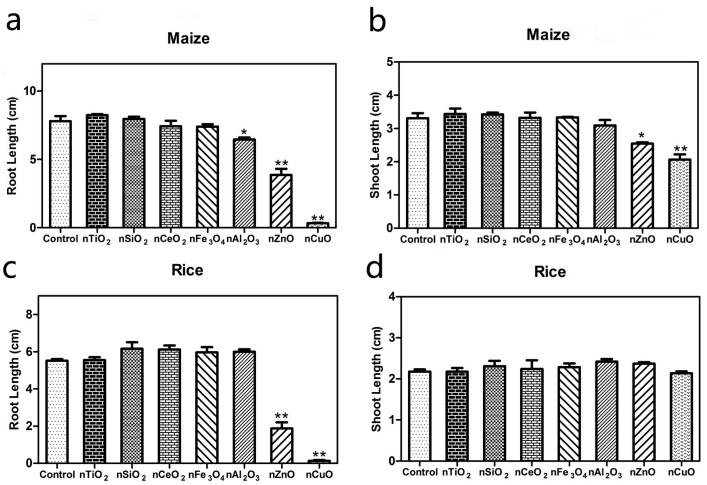
Effects of seven metal oxide NPs suspensions on (**a**) root elongation and (**b**) shoot elongation of maize at the concentration of 2000 mg·L^−1^. Effects of seven metal oxide NPs suspensions on (**c**) root elongation and (**d**) shoot elongation rice at the concentration of 2000 mg·L^−1^. The values were given as mean ± SD (standard deviation) of triplicate samples.

As shown in Figure 2 and Figure S1 (see the Supplementary Information), the effects of seven metal oxide NPs suspensions at 2000 mg·L^−1^ on root and shoot elongation of maize and rice varied among the NPs. Compared with the control, the growth of maize and rice were not affected by four metal oxide NPs: nTiO_2_, nSiO_2_, nCeO_2_ and nFe_3_O_4_. However, nCuO and nZnO reduced the root length of maize by 95.73% and 50.45%, respectively (Figure 2a). Moreover, the shoot length of maize treated with nCuO and nZnO was also reduced by 30.98% and 13.8%, as compared to the control (Figure 2b). As for nAl_2_O_3_, minor toxicity was only observed in the root of maize (*p* < 0.05), while no negative effects were observed in the shoot. As shown in Figure 2c, nCuO and nZnO reduced the root length of rice by 97.28% and 66.75%, respectively. Conversely, no inhibition effects in rice shoots were observed with nCuO and nZnO (Figure 2d). In the case of nAl_2_O_3_, no visible toxicity was observed in either the roots or shoots of rice. Due to the direct contact of radicles with the NP suspensions, the results of nCuO and nZnO both demonstrated that the toxic symptoms in roots were more serious than in shoots, which was consistent with previous studies [9,33]. Meanwhile, this result also indicated the possibility of NP uptake in the roots. To date, the uptake of different metal oxide NPs has been confirmed and electron microscopy is the primary tool and a convenient experimental methodology used in intracellular localization of NPs in plants. For instance, Wang *et al.* reported that nCuO (20–40 nm) were present in the xylem sap of maize [9]. Lin and Xing showed that nZnO (20 ± 5 nm) were present in the apoplast and protoplast of the root endodermis and stele of ryegrass [33]. 

The mechanism of metal oxide NP phytotoxicity remains unclear. It was generally reported to be related to the NP species, test plants, concentration, chemical composion, surface modification and particle size [34]. The result of preliminary assessment experiment indicated that the phytotoxicity of the metal oxide NPs varied with the species of NPs. For example, nCuO and nZnO at 2000 mg·L^−1^ showed significant inhibition on the root elongation of both maize and rice, while no obvious effects were observed with nFe_3_O_4_, nSiO_2_, nTiO_2_ and nCeO_2_. Moreover, the different effects of nAl_2_O_3_ at 2000 mg·L^−1^ on the root elongation of maize and rice proved the phytotoxicity of the metal oxide NPs varied with the test plant. 

It was well known that the pH values may affect plant growth. The experimental pHs of all the metal oxide NPs were in the range of 6.3–7.2, which should not have any negative effect on root growth [35]. In further study, three toxic NPs, (nCuO, nZnO and nAl_2_O_3_) were studied to discern the effects of the potential factors influencing the phytotoxicity: exposure concentration, ion release and particle size.

### 3.3. Dose Response Relationship of nCuO, nZnO and nAl_2_O_3_

**Figure 3:**
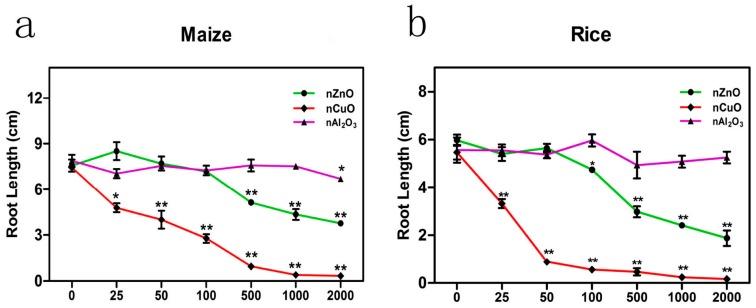
Dose-response curves of nZnO, nCuO and nAl_2_O_3_ on root growth of (**a**) maize and (**b**) rice. The values were given as mean ± SD (standard deviation) of triplicate samples with 10 seeds each.

Figure 3 and Figure S2 (see the Supplementary Information) show the effects of three metal oxide NPs (nCuO, nZnO and nAl_2_O_3_) at the concentrations of 25–2000 mg·L^−^^1^ on both maize and rice. As for nZnO, the dose-response curves were “T” shaped, and no significant inhibition effects were observed at low concentrations (100 mg·L^−1^ for maize and 50 mg·L^−1^ for rice). The root growth inhibition effects became visible with increasing concentration. However, as for nCuO, the dose-response curves present an “L” shape. Even at a low concentration of 25 mg·L^−1^, the root elongation of both maize and rice was significantly inhibited (*p* < 0.05 and *p* < 0.01, respectively). At the concentration of 1000 mg·L^−1^, the growth of maize and rice roots was almost completely terminated. Fifty percent root growth inhibitory concentrations (IC_50_) of nCuO and nZnO were estimated to be near 60 mg·L^−1^ and 1500 mg·L^−1^ for maize, and near 30 mg·L^−1^ and 400 mg·L^−1^ for rice, respectively. As for nAl_2_O_3_, the phytotoxicity was relatively low as toxicity was only observed at 2000 mg·L^−1^ in maize. The dose response relationship between metal oxide NPs and plants has been comfirmed by different studies. For example,Wang *et al.* reported that 2 mg·L^−1^ nCuO were nontoxic to maize while 10 mg·L^−1^ and 100 mg·L^−1^ nCuO significantly inhibited the root elongation [9]; Lin and Xing reported the dose response of nZnO to ryegrass [33].

### 3.4. Phytotoxicity of Released Metal Ions

**Figure 4:**
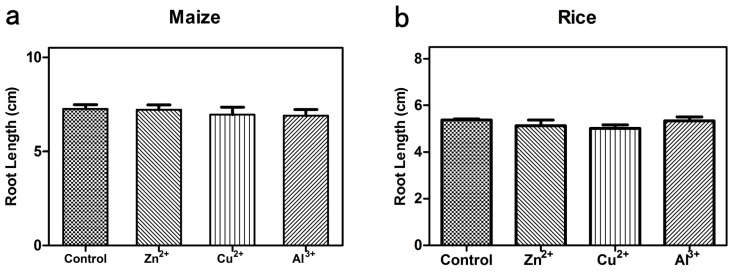
Effect of Cu^2+^, Zn^2+^ and Al^3+^ on the root elongation of (**a**) maize and (**b**) rice. The values were given as mean ± SD (standard deviation) of triplicate samples with 10 seeds each.

The release of metal ions is inevitable in metal oxide NP suspensions and it is an important interference in any study of nanotoxicity. The ICP-OES results showed that the total content of released Cu^2+^, Zn^2+^ and Al^3+^ in the corresponding metal oxide NPs suspensions were 0.11 ± 0.04 mg·L^−1^, 1.27 ± 0.03 mg·L^−1^ and 0.74 ±0.05 mg·L^−1^, respectively. In fact, these levels are a little higher than the actual released ions content since some small NPs cannot be excluded completely through centrifugation and filtration. Ion solutions were prepared from the corresponding sulfate (CuSO_4_·5H_2_O, ZnSO_4_·7H_2_O and Al_2_(SO_4_)_3_·16H_2_O ) in DI-water [36]. As shown in Figure 4, no significant inhibition effects on root growth in both maize and rice were observed in Cu^2+^, Zn^2+^ and Al^3+^ solutions compared with the control. 

Heavy metals are widely accused of being toxic to plant growth [37,38]. Many previous studies showed that metal ions (Cu^2+^ and Zn^2+^, *etc.*) could inhibit the root and stem growth of plants [39,40]. The release of metal ions is inevitable in metal oxide NP suspensions. However, most researchers have reported that no significant inhibition effects were observed in the corresponding ion solutions. For instance, Wang *et al.* reported that the phytotoxicity of nCuO mainly depends on the NP itself , but not on the Cu^2+^ released in the NP suspension [9]. Lin and Xing reported that Zn^2+^ released from the nZnO suspension did not display any phytotoxicity on radish, rape and ryegrass [41]. Consistent with these observations, our results also indicated that the amounts of metal ions released from the NPs would be negligible, probably due to the low quantity of ions released in the NP suspensions [42].

### 3.5. Effect of Particle Size on Phytotoxicity 

**Figure 5:**
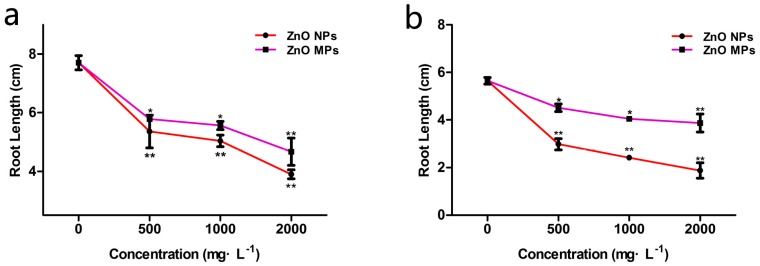
Effect of ZnO NPs and ZnO MPs on the root elongation of (**a**) maize and (**b**) rice. The values were given as mean ± SD (standard deviation) of triplicate samples with 10 seeds each.

In this study, ZnO NPs (<50 nm) and ZnO MPs (<5 μm) were selected to investigate the effect of particle size on phytotoxicity. Previous dose response relationship experiments with ZnO NPs confirmed that the concentrations of 500, 1000 and 2000 mg·L^−1^ significantly inhibited the elongation of both maize and rice roots (*p* < 0.01). Therefore, these three concentrations were selected to assess the different toxicity of ZnO NPs and ZnO MPs. As shown in Figure 5, ZnO MPs also significantly inhibited the elongation of both maize and rice roots, as compared to the controls (*p* < 0.05). However, compared with the ZnO MPs, the ZnO NPs showed greater toxicity to root elongation of both maize and rice at the three selected concentrations. For instance, compared with the control, ZnO NPs at 2000 mg·L^−1^ reduced the root length of maize and rice by 50.45% and 66.75%, while ZnO MPs only reduced the root length by 39.5% and 31.44%, respectively. The high surface/volume ratio of NPs with their smaller size make them highly reactive and with better catalytic properties, which increases their toxic potential compared with their bulk counterparts [43]. This result was in agreement with Lee *et al.* [30], who reported that ZnO NPs (44.4 ± 6.7 nm) were much more toxic than larger ZnO particles (2311 ± 304 nm) at 4000 mg·L^−1^. In addition, Lee *et al.* [42] also reported the biomass of buckwheat seedlings was more significantly reduced in response to ZnO NPs than ZnO MPs at concentrations of 10–2000 mg·L^−1^. In addition, it was reported that NPs with smaller size contained more particles for the same mass concentration and had a greater chance to penetrate through the plant cell membrane [44,45]. To date, despite the fact that various metal oxide NPs have been confirmed to be uptaken by plants [9,32,38,39], more studies are needed to test whether intracellular uptake is a requirement for causing phytotoxicity.

## 4. Conclusions 

In summary, we have reported the different phytotoxicity of seven metal oxide NPs—nCeO_2_, nFe_3_O_4_, nSiO_2_, nTiO_2_, nAl_2_O_3_, nZnO and nCuO—in maize and rice. The seed germination of maize and rice was not affected by all the seven metal oxide NPs, while the root elongation of both maize and rice were significantly inhibited by nCuO and nZnO at 2000 mg·L^−1^. nAl_2_O_3_ was only found to be slightly toxic to the root elongation of maize, while no obvious toxic effects were observed in the other four metal oxide NPs. The toxicity of nCuO, nZnO and nAl_2_O_3_ was concentration dependent in both maize and rice. No negative effects were observed in the corresponding Cu^2+^, Zn^2+^ and Al^3+^ solutions, suggesting the phytotoxicity was mainly due to the NPs themselves. In addition, ZnO NPs showed greater toxicity to root elongation of maize and rice than ZnO MPs. Overall, this study provided a unified method to test the phytotoxicity of metal oxide NPs on crop plants. Moreover, this study provided valuable information for the application of engineered NPs in agriculture and the assessment of the potential environmental risks. However, these results are basically concentrated on the phenotypic changes; the underlying molecular mechanism still needs further studies at the physiological, metabolic and genetic levels.

## Supplementary Files

Supplementary File 1
