# Supplementary material for: Assessment of the Phytotoxicity of Metal Oxide Nanoparticles on Two Crop Plants, Maize (Zea mays L.) and Rice (Oryza sativa L.)
Source: Int J Environ Res Public Health. 2015 Nov 30;12(12):15100–9. doi: 10.3390/ijerph121214963 (PMC4690899; doi:10.3390/ijerph121214963)
Supplement: Supplementary File 1 [file ijerph-12-14963-s001.pdf]

Assessment of Phytotoxicity of Metal Oxide Nanoparticles on Two Crop Plants, Maize (*Zea Mays* L.) and Rice (*Oryza Sativa* L.)

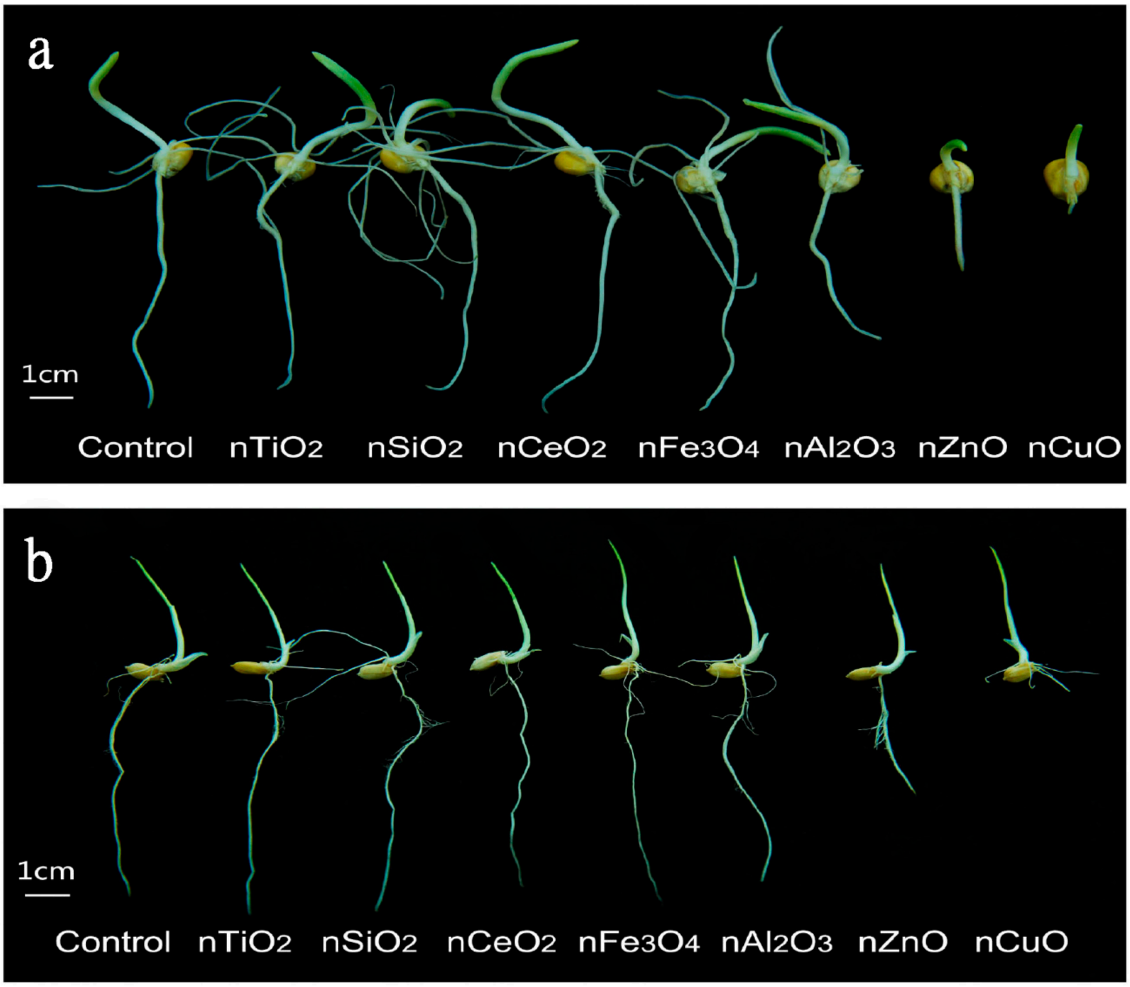

Figure S1. (**a**) Phenotype of maize seeds incubated 5 days with seven metal oxide NPs suspensions at 2000 mg·L^{-1} and without NPs. (**b**) Phenotype of rice seeds incubated 7 days with seven metal oxide NPs suspensions at 2000 mg·L^{-1} and without NPs.

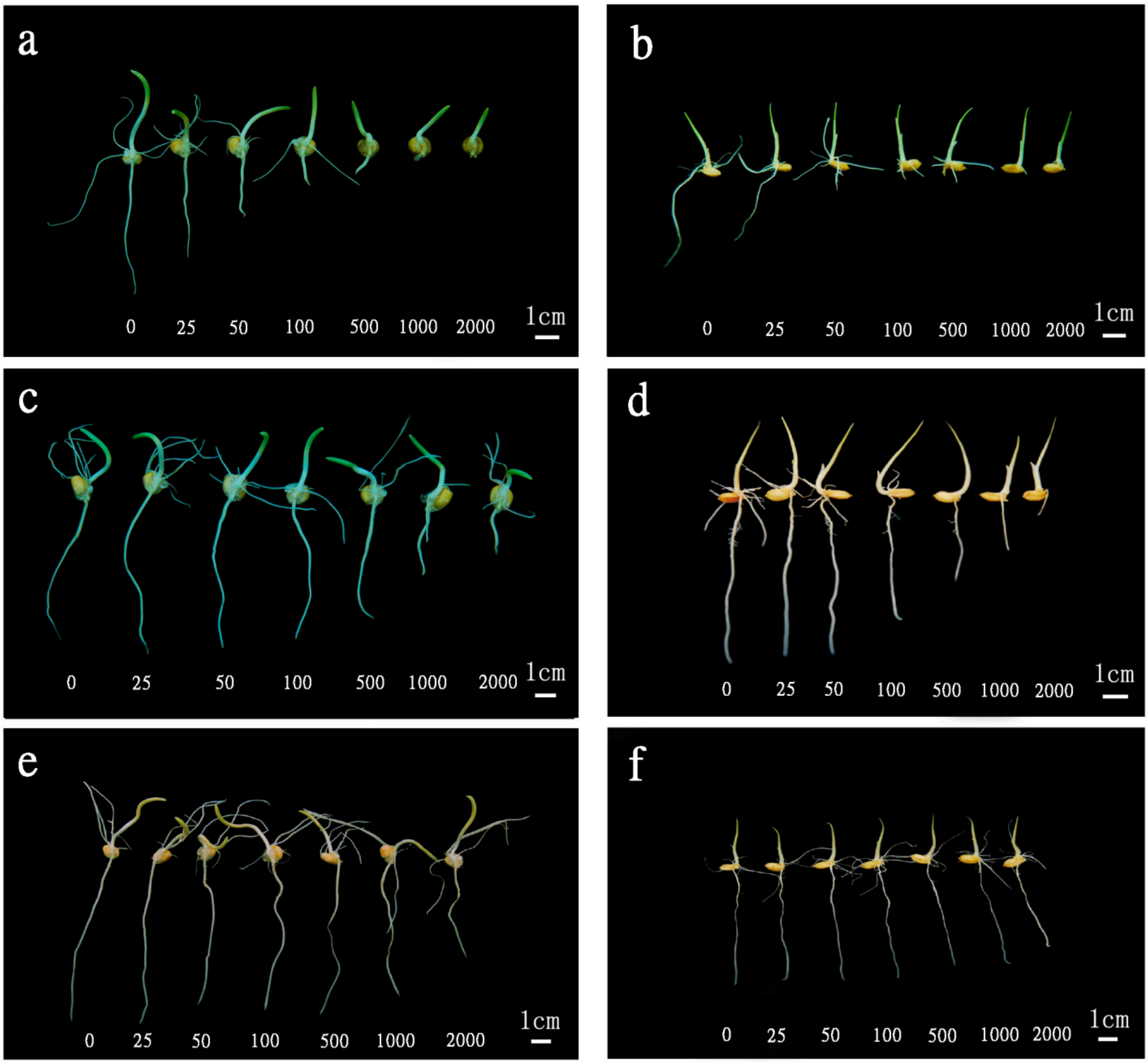

Figure S2. Phenotype of crop seeds during the germination process: (**a**) maize seeds (5 days) and (**b**) rice seeds (7 days) exposed with 0–2000 mg·L^{-1} nCuO; (**c**) maize seeds (5 days) and (**d**) rice seeds (7 days) exposed with 0–2000 mg·L^{-1} nZnO; (**e**) maize seeds (5 days) and (**f**) rice seeds (7 days) exposed with 0–2000 mg·L^{-1} nAl_2O_3.

© 2015 by the authors; licensee MDPI, Basel, Switzerland. This article is an open access article distributed under the terms and conditions of the Creative Commons Attribution license (http://creativecommons.org/licenses/by/4.0/).
